# Anticoccidial Effect of Herbal Powder “Shi Ying Zi” in Chickens Infected with *Eimeria tenella*

**DOI:** 10.3390/ani10091484

**Published:** 2020-08-24

**Authors:** Xu Song, Yunhe Li, Shufan Chen, Renyong Jia, Yongyuan Huang, Yuanfeng Zou, Lixia Li, Xinxin Zhao, Zhongqiong Yin

**Affiliations:** Natural Medicine Research Center, College of Veterinary Medicine, Sichuan Agricultural University, Chengdu 611130, China; songx@sicau.edu.cn (X.S.); lytranh@163.com (Y.L.); chensf940824@163.com (S.C.); cqrc_jry@163.com (R.J.); h415267256@163.com (Y.H.); yuanfengzou@sicau.edu.cn (Y.Z.); lilixia905@163.com (L.L.); xxinzhao@163.com (X.Z.)

**Keywords:** anticoccidial drug, coccidiosis, “Shi Yin Zi” powder

## Abstract

**Simple Summary:**

Herbal medicines are playing an increasingly important role in the control of poultry diseases. The present study demonstrated that the herbal powder “Shi Ying Zi” consisting of *Cnidium monnieri* (L.) Cuss, *Taraxacum mongolicum* Hand.-Mazz., and sodium chloride could protect chickens from infection with *Eimeria tenella* through prophylactic or therapeutic administration. The “Shi Ying Zi” powder could improve the survival rate and relative growth rate with the anti-coccidial indexes of 165 (prophylactic effect) and 144 (therapeutic effect), which were equal to positive controls (monensin and sulfamlopyrazine). The “Shi Ying Zi” powder exhibits the potential to control *E. tenella* infection.

**Abstract:**

Coccidiosis is one of the most economically important diseases affecting the poultry industry. Currently, anticoccidial drugs used in veterinary clinics show many deficiencies, and new control measures are urgently needed. This study presents an anticoccidial herbal powder “Shi Yin Zi”, which consists of *Cnidium monnieri* (L.) Cuss, *Taraxacum mongolicum* Hand.-Mazz., and sodium chloride. In chickens infected with *Eimeria tenella*, supplementation with “Shi Yin Zi” powder for 3 d prior to infection or treatment with “Shi Yin Zi” powder after infection could improve the survival rate and relative growth rate and alleviate the pathological changes in the cecum, liver, and kidney. “Shi Yin Zi” powder could recover the levels of alanine aminotransferase, creatinine, albumin, and triglycerides in serum. The hemorrhage occurrence and total number of oocysts in feces were reduced. The anti-coccidial indexes reached 165 for the prophylactic effect and 144 for the therapeutic effect. The anti-coccidial effects were equal to positive controls (monensin and sulfamlopyrazine). These results suggest that “Shi Ying Zi” powder possesses a potent anticoccidial effect and exhibits the potential to control *E. tenella* infection.

## 1. Introduction

Coccidiosis, which is caused by seven species of intracellular protozoan parasites of the genus *Eimeria*, is one of the most detrimental and lethal diseases of commercial poultry flocks [1]. It is a rapidly developing intestinal disease that presents with bloody diarrhea and listlessness and can cause high levels of mortality in affected flocks [2]. *Eimeria* oocysts are highly infectious and resilient [3]. The primary sources of the spread of oocysts are usually rodents, insects, and wild birds, and their development is favored by moisture and heat, which are not lacking in poultry farms [4]. *Eimeria* oocysts typically invade intestinal epithelium cells and cause destruction of the infected cells, resulting in a reduction of feed conversion, body weight gain, and egg production, and increased morbidity and mortality [5]. *Eimeria tenella* is one of the most frequent and destructive pathogens of the genus *Eimeria*. It parasitizes chicken cecum and induces bloody excrement and body weight reduction [6]. Therefore, the management of coccidiosis and maintenance of the immune functions for ensuring maximum performance, growth, and production in the poultry industry are fundamental requirements for profitable farming [7]. In the prevention of coccidiosis, good sanitary conditions of poultry farms, such as maintaining cleanliness, good ventilation, and the regular replacement of litter, are important for eliminating dampness and the appearance of mold, which create an ideal environment for protozoa to thrive. Furthermore, nets that prevent rodents, insects, and wild birds from entering the farms also play an important role. The regular administration of coccidiostats in feed and preventive vaccinations are typical methods used to control the disease, but coccidial oocysts gradually show drug tolerance and the production capacity of current vaccine lines is limited [8,9]. To prevent the emergence of drug resistance, new drugs have been developed and administered on a rotational basis with existing drugs. However, this has resulted in an increased cost of poultry products. Furthermore, anticoccidial drug residues in poultry products are potentially an annoyance to consumers [8]. In recent years, anticoccidial drug development has been slower than the emergence of drug-resistant *Eimeria* spp., which has stimulated the search for alternative control methods for avian coccidiosis [10].

There are many plant-derived drugs with anticoccidial effects, such as herbal extracts [11,12]. Compared with chemotherapeutic drugs, anticoccidial herbal medicines usually exhibit less drug residues and less drug resistance [13]. *Cnidium monnieri* (L.) Cuss. is a widely used traditional herbal medicine in China, Vietnam, and Japan, and its fruits have been used to treat a variety of diseases, including female vulva pain, male impotence, epilepsy, and ulcers [14]. Many phytochemicals, mainly coumarins, which are the dominant active constituents, have been identified from *C. monnieri*, and osthole is used as a quality marker in the Pharmacopoeia of China [15]. *Taraxacum mongolicum* Hand.-Mazz. is a Chinese medicine commonly used to treat infection, fever, upper respiratory tract infections, pneumonia, and other infectious diseases [16]. The phytochemical constituents of *T. mongolicum* can mainly be divided into sesquiterpenoid, triterpenoid, phytosterol, flavonoids, phenolic acids, and organic acids [17]. Caffeic acid, which is a kind of phenolic acid, is used as a quality marker in the Pharmacopoeia of China. Based on the traditional Chinese veterinary medicine theory, unlike other herbal extracts, this study prepared the herbal formula “Shi Ying Zi”, which is a powder consisting of a mixture of *C. monnieri*, *T. mongolicum*, and sodium chloride at a ratio of 55:40:5. The anticoccidial effects of “Shi Ying Zi” powder were evaluated in chickens infected with *E. tenella* through supplementation for 3 d prior to infection and administration after infection for determination of the preventive and therapeutic effects of “Shi Ying Zi” powder.

## 2. Materials and Methods

### 2.1. Preparation of “Shi Ying Zi” Powder

“Shi Ying Zi” powder consisted of *C. monnieri*, *T. mongolicum*, and sodium chloride at a ratio of 55:40:5. The two herbals were procured from Baicaotang pharmaceutical chain Co. LTD (Chengdu, China). The samples of the two plant materials were deposited at the herbarium of the Natural Medicine Research Center, College of Veterinary Medicine, Sichuan Agricultural University (Chengdu, China), with voucher numbers 2017-0168 and 2017-0205, respectively. The content of osthole in *C. monnieri* was 1.28% and the content of caffeic acid was 0.041% in *T. mongolicum* Hand-Mazz. The quality criteria of the two herbals were fitted for the standards of the Pharmacopoeia of China (Version 2015, China Pharmacopoeia Committee). The mixtures of *C. monnieri*, *T. mongolicum*, and sodium chloride were smashed into powder, followed by sieving through 150 μm ± 6.6 μm screen cloth, to give the “Shi Ying Zi” powder.

### 2.2. Oocyst

The sporulated oocysts of *E. tenella* (BJ strain) were provided by the College of Veterinary Medicine, China Agricultural University (Beijing, China) [18]. For the propagation of oocysts, twenty 14-day-old chicks free of coccidiosis were inoculated with 2 × 10^4^ sporulated oocysts of *E. tenella* by oral gavage. The cecum contents and feces from infected chicks were collected at 7 days post infection (dpi), and oocysts were separated by flotation in saturated saline, as described by Kumar (2014) [19]. The oocysts were then allowed to sporulate in 2.5% (*w*/*v*) potassium dichromate solution at 28 °C for three days, followed by washing thrice with normal saline. After the determination of the total number of oocysts by the McMaster technique, as previously described [20], the sporulated oocysts were diluted to 2 × 10^4^ oocysts/mL in normal saline.

### 2.3. Experimental Design

Two hundred 1-day-old broiler chicks free of coccidiosis were purchased from a commercial hatchery (Tianchi Poultry Co. Ltd., Mianyang, China) and reared in a sterilized poultry shed of the animal houses of Sichuan Agricultural University (Ya’an, China). The chicks were fed a ^60^Co-sterilized diet free of anticoccidial drugs (Supplementary Table S1; Xietong Biotechnology Co., Ltd., Nanjing, China). The feces were collected daily to check for oocysts [21]. At the 11th day of age, the chicks were all free of coccidiosis and were then equally divided into 10 groups (10 males and 10 females per group and each with four replicates of five chickens/cage), including an uninfected-untreated group, an infected-untreated group, four prophylactic groups (positive control group and three “Shi Ying Zi” powder groups), and four therapeutic groups (positive control group and three “Shi Ying Zi” powder groups). The chicks in the three “Shi Ying Zi” powder protective groups were prophylactically supplemented with “Shi Ying Zi” powder at doses of 5 g/kg feed (ShiYingZi-PL), 10 g/kg feed (ShiYingZi-PM), and 15 g/kg feed (ShiYingZi-PH), respectively. In the positive control group, monensin was added (100 mg/kg feed). For obtaining homogeneous mixtures of feed with “Shi Ying Zi”, the powder was firstly mixed with an equal quantity of feed, and the feed was added to the mixture at an equal quantity. The mixing process continued until all of the feed had been added. The uninfected-untreated group and infected-untreated group were fed a regular diet. At the 14th day of age, chicks, except for those in the uninfected-untreated group, were inoculated with 2 × 10^4^ sporulated oocysts of *E. tenella* by oral gavage. At 1 dpi, the chicks in the three “Shi Ying Zi” powder therapeutic groups were supplemented with “Shi Ying Zi” powder at doses of 5 g/kg feed, (ShiYingZi-TL), 10 g/kg feed (ShiYingZi-TM), and 15 g/kg feed (ShiYingZi-TH), respectively. In the positive control group, sulfachloropyrazine sodium was added to water (1 g/L).

### 2.4. Ethical Approval

All procedures involving animals and their care in this study were approved by the Ethics Committee of Sichuan Agricultural University according to the Regulation of Experimental Animal Management (State Scientific and Technological Commission of the People’s Republic of China, No. 2, 1988) and The Interim Measures of Sichuan Province Experimental Animal Management (Science and Technology Bureau of Sichuan, China, No. 25, 2013). At the end of the study (8 dpi), the chicks were anesthetized through intravenous injection (underwing vein) with sodium pentobarbital (30 mg/kg body weight) within 2 min of the birds being removed from their home pen [22]. The wing venous blood samples and tissue samples (five chicks per group) were collected. The rest of the anesthetized chicks were euthanized by manual cervical dislocation. All efforts were made to minimize the suffering of birds.

### 2.5. Clinical Symptoms

The chicks were examined daily for recording clinical signs, such as anorexia, huddling together, disheveled feathers, bloody stools, and mortality [23].

### 2.6. Bloody Diarrheal Score

The bloody diarrheal score is a qualitative estimation of the deviation of the fecal appearance from a normal state. It was obtained by scoring the bloody feces each morning from 4 to 8 dpi. The bloody diarrheal score ranged from 0 to 4: A score of 0 indicated normal feces without hemorrhage; a score of 1 indicated 1–25% hemorrhage in the feces; a score of 2 indicated 25–50% hemorrhage in the feces; a score of 3 indicated 51–75% hemorrhage in the feces; and a score of 4 indicated 76–100% hemorrhage in the feces [11].

### 2.7. Lesion Score of the Cecum

The postmortem examination was conducted 8 dpi. The dead and slaughtered birds were incised, and gross lesions in the cecum were examined by recording lesion scoring as described by Johnson and Reid [24]. The lesions included hemorrhages, thickening of the cecum wall, and mucoid discharge. The lesion score ranged from 0 to 4: A score of 0 indicated no lesion; a score of 1 indicated a mild lesion; a score of 2 indicated a moderate lesion; a score of 3 indicated a severe lesion; and a score of 4 indicated a more severe lesion.

### 2.8. Oocyst Counts

The oocyst counts in feces were measured by the McMaster method [20]. Briefly, 2 g of feces collected from 0 to 8 dpi were uniformly dispersed in 60 mL saturated salt solution with vigorous stirring, followed by filtration. Then, the filtrate was loaded onto a McMaster Egg Slide (Chongqing DiShui Experimental apparatus Co., Ltd., Chongqing, China), which has two chambers with two identical 10 mm × 10 mm × 1.5 mm grids (0.15 mL), for oocyst counting. The total number of oocysts under both of the grids was recorded. The oocyst counts (oocysts per gram) were calculated by multiplying the total number of oocysts in the two chambers by 100. The cecum contents were collected 8 dpi after postmortem examination. The oocyst counts in the cecum contents were determined as described above. The score of the oocysts index at 8 dpi ranged from 0 to 40: A score of 0 indicated the oocyst counts were 0–0.1 × 10^6^; a score of 5 indicated the oocyst counts were 0.11 × 10^6^–1 × 10^6^; a score of 10 indicated the oocyst counts were 1.1 × 10^6^–1.9 × 10^6^; a score of 20 indicated the oocyst counts were 2.0 × 10^6^–5.9 × 10^6^; a score of 30 indicated the oocyst counts were 6.0 × 10^6^–10.9 ×10^6^; and a score of 40 indicated the oocyst counts were more than 1.1 × 10^7^.

### 2.9. Anti-Coccidiosis Index

The anti-coccidiosis index (ACI) = (Relative weight gain rate + Survival rate) − (Oocysts index in cecum + Gross lesions score) [25].

The relative weight gain rate (×100%) is the ratio of the average weight gain of the infected group to the average weight gain of the uninfected-untreated group. The survival rate (×100%) is the ratio of the number of alive chicks to the total number of chicks. The average initial weight is the average weight of the chicks at 0 dpi, and the average final weight is the average weight of the chicks at 8 dpi.

When ACI is larger than 180, it means that the drug has a high efficiency; when ACI is 160–180, it means that it has a medium efficiency; when ACI is 120–160, it means that it has a low efficiency; and when ACI is lower than 120, it means that the drug does not possess anti-coccidiosis effects.

### 2.10. Biochemical Indexes

For biochemical studies, 3–4 mL wing venous blood was collected from five randomly selected chicks of each group into tubes, without any anticoagulant, at intervals of 1, 4 and 8 dpi. After clotting, serum was separated and various parameters were estimated, including total serum protein, albumin, aspartate aminotransferase, alanine aminotransferase, creatinine, and triglyceride. The globulins were calculated by subtracting the values of albumin from total serum proteins.

### 2.11. Histopathology

After dissection, the cecum, liver, and kidney tissue samples were subjected to common histological preparation. A 5 μm section of each tissue was procured and stained with hematoxylin and eosin (HE) for histopathological examination.

### 2.12. Statistical Analysis

The statistical significance was compared for the uninfected-untreated control and experimental groups by one way analysis of variance (ANOVA), followed by the Student–Newman–Keuls test, using the IBM SPSS Statistics, Version 24 program (IBM Corporation. Somers, NY, USA). The differences between groups were considered significant when values of *p* < 0.05.

## 3. Results

### 3.1. Clinical Symptoms and Mortality

After being challenged with *E. tenella*, the clinical signs and symptoms were closely monitored throughout the trial. The clinical signs, including disheveled feathers, dullness, and anorexia, were observed in the infected-untreated group at 3 dpi. The symptoms in ShiYingZi protective groups were observed at 4 dpi, which were similar to the infected-untreated group, but much milder. The mortality of the infected-untreated group was highest in all groups. The appearance of hemorrhage in feces was noted at 4 dpi in the infected-untreated group and many oocysts were found in the cecum, suggesting that the model was successfully established. For pretreatment with “Shi Ying Zi” powder, bloody stools were detected at 5 dpi.

*E. tenella* infection induced a 25% mortality rate in the infected-untreated group, which is significantly higher than the uninfected-untreated control and other infected groups. Pretreatment with “Shi Ying Zi” powder only caused a 5% mortality rate in the ShiYingZi-PL group and ShiYingZi-PH group. There was no mortality in the ShiYingZi-PM group and monensin group (Table 1). When the drugs were supplemented after infection, the mortality rates were 10% in the ShiYingZi-TL group and 5% in the ShiYingZi-TM group. In the ShiYingZi-TH group and sulfachloropyrazine sodium group, there was no mortality (Table 1). These results suggested that “Shi Ying Zi” powder and positive drugs could significantly reduce the mortality rate of chicks infected with *E. tenella*.

### 3.2. Anticoccidial Efficacy in Chickens

The bloody diarrheal score from 4 to 8 dpi (Table 2) and the total number of oocysts per gram of feces from 0 to 8 dpi (Table 3) were measured. Oocyst shedding in feces could be observed from 0 to 3 dpi. When the hemorrhagic feces were observed in the infected groups at 4 dpi, the oocyst shedding in feces could be detected, except for the ShiYingZi-PH and monensin groups. The number of oocysts in feces was decreased by ShiYingZi treatment from 4 to 8 dpi. The ShiYingZi therapeutic group (10 g/kg) exhibited a significantly decreased bloody diarrheal score in comparison with the infected-untreated control at 5 dpi. Both the prophylactic and therapeutic administration of “Shi Ying Zi” powder could significantly decrease the bloody diarrheal score in comparison with the infected-untreated control at 6 and 7 dpi. “Shi Ying Zi” powder exhibited equal potency when compared with monensin and sulfachloropyrazine sodium. These results suggested that “Shi Ying Zi” powder could alleviate the hemorrhages in feces and accelerate recovery from bloody stools. The amount of oocyst shedding in feces was also reduced.

Coccidiosis induced decreased weight gain, and the relative weight gain rate was 53% compared with the uninfected-untreated group (Table 4). The prophylactic administration of “Shi Ying Zi” powder could recover the relative weight gain rate up to 86% compared with the uninfected-untreated group, which is better than monensin. The relative weight gain rate of the “Shi Ying Zi” powder therapeutic group could reach to 77% in comparison with the infected-untreated group. The gross lesions score and cecum oocyst index were decreased after the prophylactic and therapeutic administration of “Shi Ying Zi” powder and the positive drugs. The prophylactic administration of “Shi Ying Zi” powder at the dose of 10 g/kg showed the highest anticoccidial activity, with an ACI value of 165, which is higher than that obtained for monensin (159).

### 3.3. Biochemical Indexes

The contents of total protein (Table 5), albumin (Table 6), and triglyceride (Table 7) were decreased after infection on 4 and 8 dpi (*p* < 0.05), but they were increased after pretreatment or treatment with “Shi Ying Zi” powder and positive drugs (*p* < 0.05). The serum globulin concentrations (Table 8) in the infected chicks with or without treatment were increased on 4 and 8 dpi (*p* < 0.05), and no significant difference was observed among all of the infected chicks. The contents of alanine aminotransferase (Table 9) and creatinine (Table 10) in the infected chicks were increased on 4 and 8 dpi (*p* < 0.05), and after the prophylactic and therapeutic administration of “Shi Ying Zi” powder, they were decreased (*p* < 0.05). Coccidiosis led to an increased (*p* < 0.05) content of aspartate aminotransferase on 4 and 8 dpi (Table 11), and after the prophylactic and therapeutic administration of “Shi Ying Zi” powder, it was decreased on 8 dpi (*p* < 0.05).

### 3.4. Histopathological Examination

**Figure 1:**
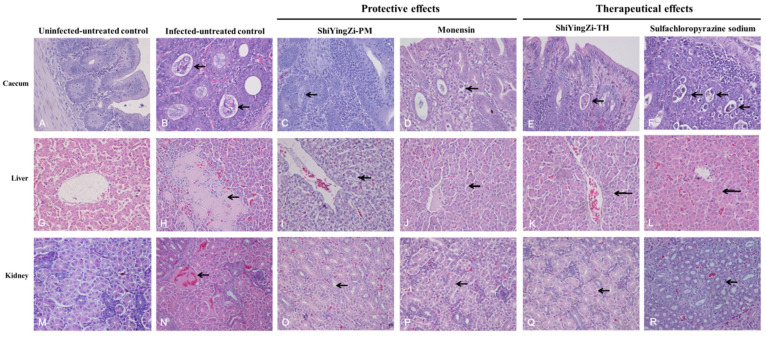
Histopathological examination (400×). In the cecum (**A**–**F**), oocysts invaded the cecum mucosa and intestinal gland ((**B**), denoted by an arrowhead) and serious karyopyknosis and necrocytosis were detected in cecum mucosa cells; in the ShiYingZi-PM group (**C**), ShiYingZi-TH group (**E**), and positive control groups ((**D**), monensin; (**F**), sulfachloropyrazine sodium), few oocysts were observed in cecum mucosa cells (denoted by an arrowhead), and the cecum mucosa cells were granular and exhibited vacuolar degeneration; (**A**) the uninfected-untreated group. In the liver (**G**–**L**), *Eimeria tenella* infection induced serious hepatocyte necrosis and inflammatory cell infiltration ((**H**), denoted by arrowhead); granular and vacuolar degeneration (denoted by arrowhead) appeared in the ShiYingZi-PM group (**I**), positive control group ((**J**), monensin; (**L**), sulfachloropyrazine sodium), and ShiYingZi-TH group (**K**); (**G**) the normal structure of the liver. In the kidney (**M**–**R**), serious granular and vacuolar degeneration and cell necrosis were observed in the infected-untreated group ((**N**), denoted by arrowhead); little granular and vacuolar degeneration (denoted by an arrowhead) was observed in the ShiYingZi-PM group (**O**), ShiYingZi-TH group (**Q**), and positive control group ((**P**), monensin; (**R**), sulfachloropyrazine sodium); in terms of the therapeutic effects, more inflammatory cell infiltration was observed in the positive control group (**R**) than in the ShiYingZi-TH group (**Q**); (**M**) the uninfected-untreated group.

In the cecum, oocysts invaded the cecum mucosa and intestinal gland (Figure 1B), and serious karyopyknosis and necrocytosis were detected in cecum mucosa cells. In the ShiYingZi-PM group (Figure 1C), ShiYingZi-TH group (Figure 1E), and positive control groups (Figure 1D, monensin; Figure 1F, sulfachloropyrazine sodium), few oocysts were observed in the cecum mucosa cells, and the cecum mucosa cells were granular and exhibited vacuolar degeneration; in the uninfected-untreated group (Figure 1A), the cecum displayed a normal structure.

In the liver, *E. tenella* infection induced serious hepatocyte necrosis and inflammatory cell infiltration (Figure 1H); granular and vacuolar degeneration appeared in the ShiYingZi-PM group (Figure 1I), ShiYingZi-TH group (Figure 1K), and positive control groups (Figure 1J, monensin; Figure 1L, sulfachloropyrazine sodium); without infection, the liver showed a normal structure (Figure 1G).

In the kidney, serious granular and vacuolar degeneration and cell necrosis were observed in the infected-untreated group (Figure 1N); mild granular and vacuolar degeneration were observed in the ShiYingZi-PM group (Figure 1O), ShiYingZi-TH group (Figure 1Q), and positive control groups (Figure 1P, monensin; Figure 1R, sulfachloropyrazine sodium); more severe inflammatory cell infiltration was observed in the sulfachloropyrazine sodium group (Figure 1R) than that in the ShiYingZi-TH group (Figure 1Q); and without infection, the kidney showed a normal structure (Figure 1I).

## 4. Discussion

Coccidiosis is one of the most economically important diseases, but commonly used anti-coccidiosis drugs often cause drug residues in meat and eggs [26]. This study provides a new herbal drug candidate called “Shi Ying Zi” for treating coccidiosis, and the potency is equal to that of monensin and sulfamlopyrazine. Unlike other herbal medicine, “Shi Ying Zi” does not need any extraction process and it is the powder of *C. monnieri*, *T. mongolicum*, and sodium chloride. Therefore, it is more financially acceptable, which is the one of the most important requirements for drugs used in the poultry industry. This study revealed that “Shi Ying Zi” powder administrated both prior to and after infection showed potent anticoccidial effects, suggesting that it could be used as a prophylactic and therapeutic drug. For prophylaxis, the recommended dosage of “Shi Ying Zi” powder is 10 g/kg; for treatment, it is 15 g/kg.

Coccidiosis usually reduces the body weight gain in broiler chicks as a result of a reduced feed intake, digestibility, and absorption of macronutrients [27]. It is accepted that weight gain is the more sensitive variable to coccidiosis and anticoccidial treatments [28]. When infective sporozoites enter the cecum mucosa by penetrating villus epithelial cells, this leads to extensive destruction of the cecum epithelium, bloody stools, and a large amount of oocyst excretion [29,30]. In this study, infected chicks were dull and depressed, exhibiting disheveled feathers and a lower feed intake, which may have been due to altered gut homeostasis that led to poor feed intake and metabolism and thus decreased weight gains [31,32]. “Shi Ying Zi” powder can alleviate the histopathological changes of the cecum, and the number of oocysts and mucosa cell necrocytosis in the cecum were decreased. Therefore, the relative weight gain rates of “Shi Ying Z” groups were also improved. The anti-coccidial index is a common index employed to evaluate the anticoccidial activity of drugs. The ACI was 165 in the “Shi Ying Z” protective group (10 g/kg), which is higher than the value for the positive control monensin. These results suggested that “Shi Ying Zi” powder is a moderate-potency anticoccidial drug.

The blood biochemical analysis reflected alterations of functional organs and some enzyme activities are often used as indicators of the site and the extent of pathological injury [33,34]. The infection of coccidiosis can affect the body’s liver function, which leads to a decrease of the triglyceride content in the serum [7]. The nutrient malabsorption, hepatocellular damage, hemorrhagic enteritis, kidney dysfunction, and inappetence might lead to a decrease of the serum albumin and total protein content [31,35] and an increase of the alanine aminotransferase and aspartate amino transferase contents in the serum [36]. Similarly, there was a significant decrease in the contents of albumin, total protein, and triglyceride and a significant increase in the contents of alanine aminotransferase and aspartate amino transferase after infection. After prophylaxis or treatment with “Shi Ying Zi” powder, these changed biochemical parameters were recovered towards normal levels. In the histopathological study, serious liver damage after *E. tenella* infection could be observed, while “Shi Ying Zi” powder could alleviate these symptoms. These results suggested that coccidiosis could affect the body’s liver function, and “Shi Ying Zi” powder could protect and alleviate the liver damage.

Creatinine is a metabolite of muscle creatine and phosphate, which is not affected by the food protein content and protein metabolism. The content of creatinine in the body is relatively constant and only reflected by the renal clearance and renal function [37,38]. In the case of renal insufficiency, creatinine accumulates in the body and becomes a toxin that is harmful to the body, so the creatinine content can respond to the damage to renal parenchyma [39]. There was a significant increase in creatinine after infection, which was consistent with a previous report [40]. The content of creatinine in the ShiYingZi-treated group was significantly lower. These results showed that “Shi Ying Zi” powder could protect the kidney damage caused by coccidiosis.

Traditional Chinese medicine (TCM) formulas usually exhibit numerous pharmacological effects due to multiple components and targets [41]. It has been revealed that a *T. mongolicum* extract could inhibit coccidian oocyst sporulation [42] and reduce liver injury induced by alcohol and environmental pollutants [43,44]. Chlorogenic acid and caffeic acid, which are the main components of *T. mongolicum*, also contributed to the anticoccidial efficacy of a herbal formula [19]. In China, *C. monnieri* is used to treat coccidiosis in many TCM prescriptions [45] and can improve the kidney function in kidney yang deficient mice [14]. Osthole, which is the main active constituent, is also beneficial for fatty liver [46]. The herbal formula called “Shi Ying Zi” presented in this study showed multidirectional activity, which may be attributed to the multiple components and biological activities of *C. monnieri* and *T. mongolicum*, such as chlorogenic acid, caffeic acid, and osthole. Further study should be conducted to find out the anticoccidial constituents of “Shi Ying Zi” and explore the anticoccidial mechanism.

## 5. Conclusions

The traditional Chinese medicine formula “Shi Ying Zi” powder can prevent and treat *E. tenella* infection in broiler chickens. “Shi Ying Zi” powder could be an alternative control measure for *E. tenella* infection.

## Figures and Tables

**Table 1:** Mortality in different groups from 4 to 8 days post infection (dpi).

Groups	4 dpi	5 dpi	6 dpi	7 dpi	8 dpi	Total Mortality
Uninfected-untreated control	0	0	0	0	0	0 ^a^
Infected-untreated control	2	2	1	0	0	5 ^b^
ShiYingZi-PL	0	1	0	0	0	1 ^ac^
ShiYingZi-PM	0	0	0	0	0	0 ^a^
ShiYingZi-PH	1	0	0	0	0	1 ^ac^
Monensin	0	0	0	0	0	0 ^a^
ShiYingZi-TL	1	1	0	0	0	2 ^ac^
ShiYingZi-TM	0	1	0	0	0	1 ^ac^
ShiYingZi-TH	0	0	0	0	0	0 ^a^
Sulfachloropyrazine sodium	0	0	0	0	0	0 ^a^

^a,b,c^ Different letters for the total mortality indicate that a significant difference existed (*p* < 0.05).

**Table 2:** The bloody diarrheal score from 4 to 8 dpi.

Groups	4 dpi	5 dpi	6 dpi	7 dpi	8 dpi
Uninfected-untreated control	0 ^a^	0 ^a^	0 ^a^	0 ^a^	0 ^a^
Infected-untreated control	1 ^a^	2 ^b^	3 ^b^	3 ^b^	1 ^a^
ShiYingZi-PL	1 ^a^	2 ^b^	3 ^b^	2 ^c^	1 ^a^
ShiYingZi-PM	0 ^a^	2 ^b^	2 ^c^	2 ^c^	0 ^a^
ShiYingZi-PH	1 ^a^	2 ^b^	3 ^b^	3 ^b^	0 ^a^
Monensin	0 ^a^	2 ^b^	2^c^	2 ^c^	0 ^a^
ShiYingZi-TL	0 ^a^	2 ^b^	3 ^b^	2 ^c^	0 ^a^
ShiYingZi-TM	0 ^a^	1 ^a^	2 ^c^	1 ^a^	1 ^a^
ShiYingZi-TH	1 ^a^	2 ^b^	2 ^c^	2 ^c^	0 ^a^
Sulfachloropyrazine sodium	0 ^a^	1 ^a^	2 ^c^	1 ^a^	0 ^a^

^a,b,c^ Different letters indicate that a significant difference existed (*p* < 0.05).

**Table 3:** The number of oocysts per gram of feces (×10^6^).

Groups	4 dpi	5 dpi	6 dpi	7 dpi	8 dpi
Uninfected-untreated control	0	0	0	0	0
Infected-untreated control	0.27	2.78	1.93	1.54	1.15
ShiYingZi-PL	0.09	2	1.71	0.85	0.55
ShiYingZi-PM	0.14	2.13	1.24	1.03	0.60
ShiYingZi-PH	0	3.12	1.75	1.06	0.83
Monensin	0	1.56	1.69	0.75	0.42
ShiYingZi-TL	0.21	2.17	1.78	1.13	0.90
ShiYingZi-TM	0.33	2.33	1.51	1.23	0.95
ShiYingZi-TH	0.25	2.49	1.88	1.07	0.46
Sulfachloropyrazine sodium	0.19	2.12	1.25	0.99	0.78

**Table 4:** Anti-coccidiosis index.

Indicators	Uninfected-Untreated Control	Infected-Untreated Control	Protective Effect				Therapeutic Effect			
			ShiYingZi-PL	ShiYingZi-PM	ShiYingZi-PH	Monensin	ShiYingZi-TL	ShiYingZi-TM	ShiYingZi-TH	Sulfachloropyrazine Sodium
Average initial weight/g	165.27 ± 20.5	157.07 ± 25.1	164.07 ± 20.72	155.13 ± 14.17	160.43 ± 14.98	146.33 ± 12.56	160.13 ± 18.12	152.4 ± 18.72	149.33 ± 19.29	159.2 ± 19.93
Average final weight/g	225 ± 26.06 ^b^	189.14 ± 29.44 ^a^	210.27 ± 22.29 ^ab^	204.82 ± 18.07 ^ab^	209.41 ± 16.57 ^ab^	191.6 ± 14.9 ^a^	204 ± 21.01 ^a^	188.73 ± 23.08 ^a^	189.91 ± 23.4 ^a^	207.44 ± 17.9 ^ab^
Relative weight gain rate (%)	100	53	78	86	83	78	74	71	77	80
Survival rate (%)	100	75	95	100	95	100	90	95	100	100
Gross lesions score	0 ± 0 ^a^	35 ± 5.27 ^c^	14 ± 11.74 ^b^	11 ± 9.94 ^b^	14 ± 11.74 ^b^	9 ± 8.76 ^b^	19 ± 13.7 ^b^	15 ± 10.8 ^b^	13 ± 4.83 ^b^	11 ± 7.38 ^b^
Cecum oocyst index	0	40	20	10	10	10	20	20	20	10
ACI	-	-	139	165	154	159	125	131	144	159
Therapeutic evaluation	-	-	low	medium	low	low	low	low	low	low

^a,b,c^ Significant differences exist when there are different letters in each column (*p* < 0.05). *n* = 20. The initial weight was the weight on the day of infection and the final weight was the weight at 8 dpi.

**Table 5:** Total protein content (g/L).

Groups	1 dpi	4 dpi	8 dpi
Uninfected-untreated control	24.56 ± 1.43 ^a^	25.26 ± 1.16 ^c^	25.48 ± 1.23 ^b^
Infected-untreated control	25.48 ± 2.35 ^a^	21.58 ± 1^a^	20.94 ± 0.98 ^a^
ShiYingZi-PL	24.8 ± 1.02 ^a^	23.52 ± 0.82 ^b^	27.4 ± 2.33 ^bc^
ShiYingZi-PM	23.66 ± 2.19 ^a^	24.28 ± 1.01 ^bc^	28.62 ± 1.42 ^c^
ShiYingZi-PH	24.3 ± 0.92 ^a^	25.22 ± 1.35 ^c^	28.08 ± 1.98 ^c^
Monensin	23.6 ± 1.98 ^a^	23.64 ± 1.43 ^b^	28.14 ± 0.8 ^c^
ShiYingZi-TL	23.67 ± 2.23 ^a^	20.67 ± 1.72 ^a^	24.13 ± 1.43 ^b^
ShiYingZi-TM	23.1 ± 0.95 ^a^	21.73 ± 1.40 ^a^	23.63 ± 1.68 ^b^
ShiYingZi-TH	24.40 ± 0.92 ^a^	21.47 ± 3.00 ^a^	25.23 ± 1.22 ^b^
Sulfachloropyrazine sodium	24.27 ± 3.40 ^a^	22.67 ± 0.57 ^a^	25.80 ± 2.19 ^b^

^a,b,c^ Significant differences exist when there are different letters in each column (*p* < 0.05). *n* = 5.

**Table 6:** The albumin content (g/L).

Groups	1 dpi	4 dpi	8 dpi
Uninfected-untreated control	11.78 ± 0.79 ^a^	12.34 ± 0.84 ^c^	13.74 ± 1.03 ^c^
Infected-untreated control	11.78 ± 1.11 ^a^	4.64 ± 0.61 ^a^	4.82 ± 1.36 ^a^
ShiYingZi-PL	10.9 ± 1.51 ^a^	6.1 ± 0.68 ^b^	10.42 ± 0.87 ^b^
ShiYingZi-PM	10.48 ± 2.02 ^a^	7.44 ± 0.8 ^b^	11.04 ± 0.87 ^b^
ShiYingZi-PH	11.3 ± 0.89 ^a^	7.24 ± 1.39 ^b^	11.26 ± 0.63 ^b^
Monensin	10.8 ± 0.99 ^a^	6.6 ± 1.44 ^b^	11.46 ± 1.33 ^b^
ShiYingZi-TL	10.73 ± 1.58 ^a^	4.10 ± 0.92 ^a^	8.40 ± 0.92 ^b^
ShiYingZi-TM	10.90 ± 0.56 ^a^	4.57 ± 0.61 ^a^	7.80 ± 0.61 ^b^
ShiYingZi-TH	11.67 ± 1.80 ^a^	6.00 ± 1.25 ^a^	7.20 ± 1.25 ^b^
Sulfachloropyrazine sodium	11.53 ± 2.87 ^a^	6.20 ± 1.00 ^a^	9.43 ± 1.00 ^b^

^a,b,c^ Significant differences exist when there are different letters in each column (*p* < 0.05). *n* = 5.

**Table 7:** Triglyceride content (mmol/L).

Groups	1 dpi	4 dpi	8 dpi
Uninfected-untreated control	0.57 ± 0.06 ^a^	0.59 ± 0.04 ^c^	0.57 ± 0.07 ^c^
Infected-untreated control	0.57 ± 0.08 ^a^	0.38 ± 0.04 ^a^	0.34 ± 0.05 ^a^
ShiYingZi-PL	0.56 ± 0.03 ^a^	0.44 ± 0.03 ^b^	0.47 ± 0.03 ^b^
ShiYingZi-PM	0.54 ± 0.05 ^a^	0.49 ± 0.04 ^b^	0.48 ± 0.03 ^b^
ShiYingZi-PH	0.56 ± 0.07 ^a^	0.47 ± 0.04 ^b^	0.47 ± 0.04 ^b^
Monensin	0.57 ± 0.06 ^a^	0.48 ± 0.05 ^b^	0.45 ± 0.04 ^b^
ShiYingZi-TL	0.55 ± 0.05 ^a^	0.39 ± 0.04 ^a^	0.40 ± 0.06 ^ab^
ShiYingZi-TM	0.54 ± 0.06 ^a^	0.36 ± 0.03 ^a^	0.41 ± 0.03 ^ab^
ShiYingZi-TH	0.53 ± 0.09 ^a^	0.38 ± 0.07 ^a^	0.46 ± 0.04 ^b^
Sulfachloropyrazine sodium	0.56 ± 0.05 ^a^	0.37 ± 0.04 ^a^	0.45 ± 0.04 ^b^

^a,b,c^ Significant differences exist when there are different letters in each column (*p* < 0.05). *n* = 5.

**Table 8:** The globulin content (g/L).

Groups	1 dpi	4 dpi	8 dpi
Uninfected-untreated control	12.78 ± 0.85 ^a^	12.92 ± 0.69 ^a^	11.76 ± 0.87 ^a^
Infected-untreated control	12.78 ± 0.86 ^a^	16.94 ± 1.38 ^b^	16.10 ± 0.58 ^b^
ShiYingZi-PL	13.9 ± 2.09 ^a^	17.42 ± 0.53 ^b^	16.98 ± 1.65 ^b^
ShiYingZi-PM	13.18 ± 1.45 ^a^	16.84 ± 0.3 ^b^	17.58 ± 0.89 ^b^
ShiYingZi-PH	13.00 ± 0.74 ^a^	17.96 ± 0.8 ^b^	16.82 ± 1.5 ^b^
Monensin	12.8 ± 1.31 ^a^	17.04 ± 0.78 ^b^	16.68 ± 0.9 ^b^
ShiYingZi-TL	12.93 ± 1.77 ^a^	16.57 ± 2.57 ^b^	15.73 ± 1.65 ^b^
ShiYingZi-TM	12.20 ± 0.46 ^a^	17.17 ± 0.83 ^b^	15.83 ± 2.94 ^b^
ShiYingZi-TH	12.70 ± 1.97 ^a^	15.47 ± 1.76 ^b^	18.03 ± 0.80 ^b^
Sulfachloropyrazine sodium	12.73 ± 1.83 ^a^	16.47 ± 1.56 ^b^	16.37 ± 0.86 ^b^

^a,b,c^ Significant differences exist when there are different letters in each column (*p* < 0.05). *n* = 5.

**Table 9:** The alanine aminotransferase content (U/L).

Groups	1 dpi	4 dpi	8 dpi
Uninfected-untreated control	4.18 ± 0.31 ^a^	4.08 ± 0.22 ^a^	4.14 ± 0.23 ^a^
Infected-untreated control	4.08 ± 0.53 ^a^	5.6 ± 0.5 ^b^	6.06 ± 0.21 ^c^
ShiYingZi-PL	4.1 ± 0.37 ^a^	4.66 ± 0.15 ^a^	4.78 ± 0.23 ^b^
ShiYingZi-PM	4.34 ± 0.49 ^a^	4.64 ± 0.32 ^a^	4.34 ± 0.15 ^a^
ShiYingZi-PH	4.14 ± 0.45 ^a^	4.66 ± 0.21 ^a^	4.34 ± 0.15 ^a^
Monensin	4.16 ± 0.54 ^a^	4.5 ± 0.25 ^a^	4.24 ± 0.26 ^a^
ShiYingZi-TL	4.30 ± 0.78 ^a^	5.53 ± 0.32 ^b^	5.13 ± 0.15 ^b^
ShiYingZi-TM	4.16 ± 0.35 ^a^	5.16 ± 0.67 ^b^	4.93 ± 0.35 ^b^
ShiYingZi-TH	4.57 ± 0.61 ^a^	5.33 ± 0.41 ^b^	4.80 ± 0.60 ^a^
Sulfachloropyrazine sodium	4.60 ± 0.35 ^a^	5.03 ± 067 ^b^	4.83 ± 0.60 ^a^

^a,b,c^ Significant differences exist when there are different letters in each column (*p* < 0.05). *n* = 5.

**Table 10:** The creatinine content (μmol/L).

Groups	1 dpi	4 dpi	8 dpi
Uninfected-untreated control	18.02 ± 2.27 ^a^	17.48 ± 1.28 ^a^	17.08 ± 2.16 ^a^
Infected-untreated control	17.98 ± 2.16 ^a^	26.62 ± 3.46 ^c^	28 ± 3.17 ^c^
ShiYingZi-PL	18.34 ± 1.4 ^a^	23.1 ± 3.49 ^b^	22.36 ± 1.67 ^b^
ShiYingZi-PM	18.34 ± 1.78 ^a^	22.18 ± 2.04 ^b^	20.98 ± 1.2 ^b^
ShiYingZi-PH	18.18 ± 2.53 ^a^	21.4 ± 1.78 ^b^	21.36 ± 1.53 ^b^
Monensin	18.52 ± 1.43 ^a^	23.42 ± 1.54 ^b^	21.04 ± 1.23 ^b^
ShiYingZi-TL	17.50 ± 1.90 ^a^	25.03 ± 1.77 ^b^	22.73 ± 1.71 ^b^
ShiYingZi-TM	19.13 ± 1.72 ^a^	24.86 ± 3.22 ^b^	23.66 ± 1.59 ^b^
ShiYingZi-TH	18.56 ± 2.00 ^a^	24.46 ± 1.60 ^b^	23.70 ± 1.83 ^b^
Sulfachloropyrazine sodium	19.76 ± 1.04 ^a^	25.40 ± 2.52 ^b^	21.56 ± 2.33 ^b^

^a,b,c^ Significant differences exist when there are different letters in each column (*p* < 0.05). *n* = 5.

**Table 11:** The aspartate aminotransferase content (U/L).

Groups	1 dpi	4 dpi	8 dpi
Uninfected-untreated control	220.46 ± 11.77 ^a^	223.22 ± 12.06 ^a^	230.62 ± 23.95 ^a^
Infected-untreated control	222.56 ± 14.18 ^a^	263.46 ± 9.28 ^b^	275.9 ± 10.79 ^c^
ShiYingZi-PL	216.3 ± 13.94 ^a^	256.66 ± 11.08 ^b^	259.28 ± 7.49 ^b^
ShiYingZi-PM	214.38 ± 17.06 ^a^	254.54 ± 7.25 ^b^	250.12 ± 5.36 ^b^
ShiYingZi-PH	214.1 ± 14.39 ^a^	251.3 ± 7.11 ^b^	251.32 ± 12.03 ^b^
Monensin	224.34 ± 8.5 ^a^	263.3 ± 11.9 ^b^	253.08 ± 6.31 ^b^
ShiYingZi-TL	224.20 ± 9.78 ^a^	258.33 ± 10.69 ^b^	264.03 ± 9.18 ^b^
ShiYingZi-TM	232.33 ± 8.88 ^a^	263.73 ± 16.22 ^b^	267.37 ± 20.15 ^b^
ShiYingZi-TH	220.50 ± 11.05 ^a^	264.50 ± 9.97 ^b^	264.43 ± 21.63 ^b^
Sulfachloropyrazine sodium	230.50 ± 9.10 ^a^	263.43 ± 19.90 ^b^	254.07 ± 0.36 ^b^

^a,b,c^ Significant differences exist when there are different letters in each column (*p* < 0.05). *n* = 5.

## Supplementary Materials

The following are available online at https://www.mdpi.com/2076-2615/10/9/1484/s1, Table S1: Nutritional components of the diet.
