# Supplementary material for: Anticoccidial Effect of Herbal Powder “Shi Ying Zi” in Chickens Infected with Eimeria tenella
Source: Animals (Basel). 2020 Aug 24;10(9):1484. doi: 10.3390/ani10091484 (PMC7552158; doi:10.3390/ani10091484)
Supplement: Supplementary file 1 [file animals-10-01484-s001.pdf]

Supplementary Table 1. Nutritional components of the diet

Components [a]	Contents (%)
Corn	61.6
Bean flour	24.7
Wheat bran	3.0
Fish meal	6.0
Vegetal oil	1.5
Additive [b]	3.2

[a] the feed is consisted of crude protein (21.54%), Ca (1%), P (0.65%), Lys (1.56%), and salt (0.37%).
[b] the composition of the additive (per kg): 240mg of copper sulfate, 466mg of ferrous sulfate, 360mg of zinc sulfate, 80mg of manganese sulfate, 0.8mg of potassium iodide, 0.42mg of cobalt chloride, 9,800mg of limestone, 11,600mg of dicalcium phosphate, 150mg of vitamin, 350mg of choline chloride, and 1,600mg of methionine.
